# The Possible Effects of Galectin-3 on Mechanisms of Renal and Hepatocellular Injury Induced by Intravascular Hemolysis

**DOI:** 10.3390/ijms25158129

**Published:** 2024-07-25

**Authors:** Mirjana Grujcic, Marija Milovanovic, Jelena Nedeljkovic, Danijela Jovanovic, Dragana Arsenijevic, Natalija Solovjova, Vesna Stankovic, Irena Tanaskovic, Aleksandar Arsenijevic, Jelena Milovanovic

**Affiliations:** 1 Institute for Transfusiology and Hemobiology of Military Medical Academy, 11000 Belgrade, Serbia; mirjanamilinkovic4@gmail.com; 2 Center for Harm Reduction of Biological and Chemical Hazards, Faculty of Medical Sciences, University of Kragujevac, Svetozara Markovića 69, 34000 Kragujevac, Serbia; marijaposta@gmail.com (M.M.); menki@hotmail.rs (D.A.); wesna.stankovic@gmail.com (V.S.); salvatoredjulijano@gmail.com (A.A.); 3 Department of Microbiology and Immunology, Faculty of Medical Sciences, University of Kragujevac, Svetozara Markovića 69, 34000 Kragujevac, Serbia; 4 Department of Medical Statistics and Informatics, Faculty of Medical Sciences, University of Kragujevac, 34000 Kragujevac, Serbia; jelena.dimitrijevic10@gmail.com; 5 Department of Internal Medicine, Faculty of Medical Sciences, University of Kragujevac, 34000 Kragujevac, Serbia; daziv81@yahoo.com; 6 Department of Pharmacy, Faculty of Medical Sciences, University of Kragujevac, 34000 Kragujevac, Serbia; 7 Academy of Applied Studies Belgrade, The College of Health Science, Cara Dušana 254, 11080 Belgrade, Serbia; natalijas-63@yandex.ru; 8 Department of Pathology, Faculty of Medical Sciences, University of Kragujevac, 34000 Kragujevac, Serbia; 9 Department of Histology and Embriology, Faculty of Medical Sciences, University of Kragujevac, 34000 Kragujevac, Serbia

**Keywords:** galectin-3, intravascular hemolysis, kidney damage, liver damage

## Abstract

Intravascular hemolysis is a central feature of congenital and acquired hemolytic anemias, complement disorders, infectious diseases, and toxemias. Massive and/or chronic hemolysis is followed by the induction of inflammation, very often with severe damage of organs, which enhances the morbidity and mortality of hemolytic diseases. Galectin-3 (Gal-3) is a β-galactoside-binding lectin that modulates the functions of many immune cells, thus affecting inflammatory processes. Gal-3 is also one of the main regulators of fibrosis. The role of Gal-3 in the development of different kidney and liver diseases and the potential of therapeutic Gal-3 inhibition have been demonstrated. Therefore, the objective of this review is to discuss the possible effects of Gal-3 on the process of kidney and liver damage induced by intravascular hemolysis, as well as to shed light on the potential therapeutic targeting of Gal-3 in intravascular hemolysis.

## 1. Introduction

Intravascular hemolysis is a process that characterizes numerous pathological conditions including autoimmune hemolytic anemia (AIHA), sickle cell disease (SCD), thalassemia, spherocytosis, paroxysmal nocturnal hemoglobinuria (PNH), thrombotic microangiopathies, infections (malaria), mechanical heart valve-induced anemia, chemical-induced anemias, and others. The intravascular destruction of red cells results in the release of heme and hemoglobin, components of erythrocytes with proinflammatory and pro-oxidative effects when found extracellularly. Several protective mechanisms, including the action of scavenging molecules, haptoglobin and hemopexin, and the enzyme (heme oxygenase-1) HO-1, prevent free heme and hemoglobin from damaging tissue structures. Haptoglobin and hemopexin bind heme and hemoglobin, respectively, and transport them into the liver and spleen in a stable, nonreactive state [1,2], while HO-1 mediates reaction of heme degradation [3]. However, when protective mechanisms are overwhelmed, as in cases of massive hemolysis, systemic damage is inevitable [4]. The toxic mechanisms of hemoglobin and heme are based on their pro-oxidative capacity, interactions with complement and coagulation cascades, and ability to induce inflammation [5]. Heme also acts as a damage-associated molecular pattern (DAMP) and, thus, stimulates the cells of innate immunity [6,7]. The organs most affected by intravascular hemolysis are the kidneys, but the liver and spleen are also affected because the metabolism of free heme and hemoglobin continues in these organs after their transport by scavenger proteins [8].

Galectin-3 (Gal-3), a β-galactoside-binding protein that binds carbohydrates through its carbohydrate recognition domain, is produced by diverse types of cells and can have both pro- and anti-inflammatory effects depending on cell compartment and type of target cell [9]. It has several proinflammatory actions while developing immune responses to microbes but also acts as a DAMP molecule [10,11]. It is associated with tissue fibrosis and is known as a marker of fibrosis and organ failure [12]. Although the contribution of Gal-3 to the pathophysiologies of various diseases is well known, the role of Gal-3 in the process of intravascular hemolysis and organ damage induced by heme and hemoglobin has never been examined. There are implications that both heme and Gal-3 contribute to the induction of inflammation in a similar way, namely by binding the same innate immune receptors and activating inflammasomes, resulting in the recruitment of immune cells. The aim of this review is, according to known mechanisms of kidney and liver injuries induced by acute intravascular hemolysis and results obtained from investigation of the role of Gal-3 in the development of different inflammatory and fibrotic renal and liver diseases, to give a relevant rationale for a possible role of Gal-3 in the pathogenesis of intravascular hemolysis-induced organ damage [5,12,13,14,15,16].

## 2. Gal-3: Structure and Function

**Figure 1:**
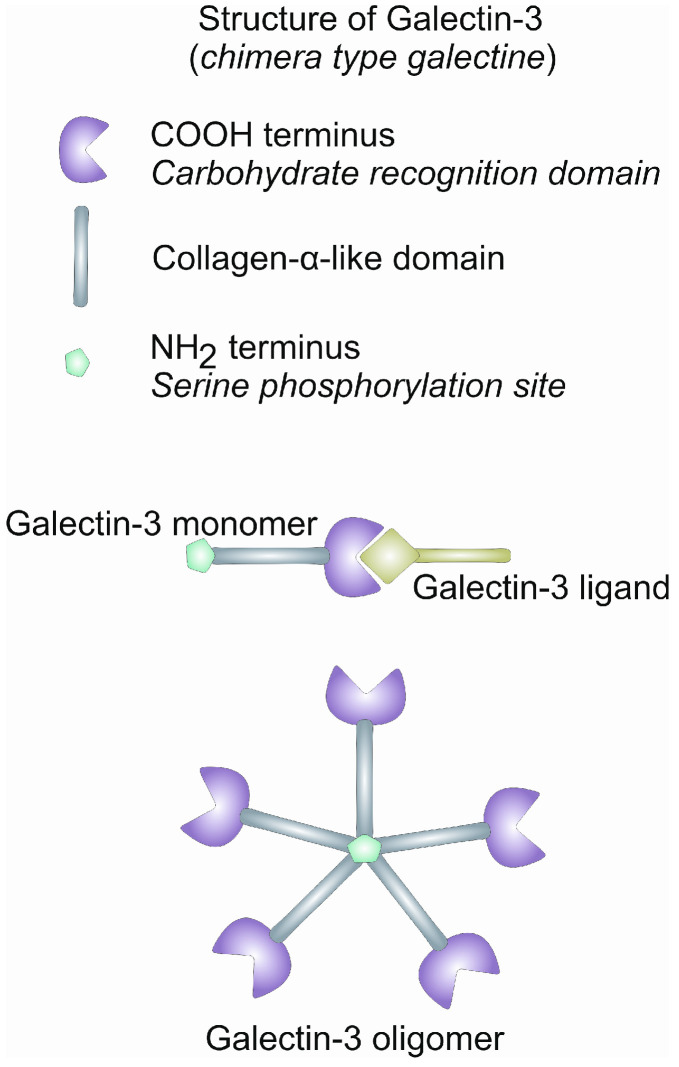
Structure of Gal-3. Schematic presentation of Gal-3 monomer and oligomerization.

Galectins are a family of evolutionarily conserved proteins that are initially recognized as β-galactoside-binding proteins. There are 15 different galectins known to exist in Mammals, all of which contain one or two conserved carbon recognition domains (CRDs) [17]. These molecules can be found in intracellular and extracellular compartments [18]. Some galectins have a wide tissue distribution, while others are specific for certain tissues [9]. Galectins can be divided into three groups: (1) prototype—this contains single CRDs (Gal-1, Gal-2, Gal-5, Gal-7, Gal-10, Gal-11, Gal-13, Gal-14, and Gal-15); (2) tandem repeats—this contain two CRDs (Gal-4, Gal-6, Gal-8, Gal-9, and Gal-12); and (3) chimera—this has only one member, Gal-3, which consists of an extended N-terminal domain and one CRD (Figure 1) [19]. The N-terminal domain enables the oligomerization of Gal-3 and pentamer formation and provides stabilization of the whole molecule (Figure 1) [20]. Also, the N-terminal domain can bind and interact with other intracellular proteins and is prone to proteolysis by matrix metalloproteinases [13,21]. Between the N-terminal domain and CRD is located a collagen-like domain, rich in proline, alanine, and glycine, that supports the oligomerization of Gal-3 and its interaction with numerous proteins present at the cell membrane, in the extracellular matrix, in biological fluids and intracellularly [22,23]. Gal-3 is mostly localized in the cytosol but can also be found in the nucleus, and it can be secreted to an extracellular compartment by a not-well-defined non-classical secretory pathway that involves neither the Golgi system nor the endoplasmatic reticulum [24]. In the extracellular environment, Gal-3 can also bind galactose-containing glycoproteins and glycolipids, polymerize and form lattices that can crosslink glycosylated ligands, and regulate the movement of plasma membrane glycoproteins and glycolipids [25,26]. Functions of Gal-3 depend on its localization. The main role of Gal-3 in the cytosol is the promotion of cell survival and proliferation and anti-apoptotic activity [27,28,29,30]. In the nucleus, Gal-3 promotes pre-mRNA splicing by incorporation into spliceosomes and regulates the transcription of genes [30].

Extracellular Gal-3 modulates interactions between the cells, as well as between the cells and components of the extracellular matrix, like fibronectin, laminin, chondroitin sulfate, tenascin, the Mac-2 binding protein, and integrins, thus affecting the adhesion of cells to endothelium and cell migration [31,32,33,34]. Gal-3 has wide expression throughout all human tissues: immune cells like macrophages, monocytes, dendritic cells, activated T and B lymphocytes, NK cells, mast cells, eosinophils, epithelial cells, endothelial cells, fibroblasts, etc. [35,36]. Gal-3 is known to have a more specific pattern of expression during embryonic development, when is dominantly found in epithelial cells, kidneys, chondrocytes, myocardial cells and liver. During embryogenesis Gal-3 navigates factors important for the proper polarization and migration of cells [37]. However, Gal-3 knock-out mice do not show any obvious signs of abnormality, except premature cellular senescence without oncogenic stress [38]. In adult mice, Gal-3 shows significant and selective expression in derivatives of uretic bud and cloaca, and it seems to play a role in the development of complex kidney tissue structures [39]. Gal-3 seems to be involved in the homeostasis of normal tubules, but not glomerular cells, even though many pathological stimuli can lead to changes in Gal-3 expression [14,40]. Normal hepatocytes do not express Gal-3, but its expression is upregulated in various inflammatory states such as liver cirrhosis and hepatocellular carcinoma [41].

## 3. Gal-3 and the Innate Immunity

Gal-3 plays diverse and often contradictory roles in innate immune responses, depending on the type of cell observed, but its role is also dependent on whether is found in the intracellular or extracellular compartment. Interactions between carbohydrate binding proteins (CBPs) and glycoconjugates are found to be more complex and less predictable when compared to protein receptor–ligand signaling [13]. Many studies examined the role of Gal-3 in antigen presenting cells, which are the first cells that encounter foreign antigens or recognize DAMPs and give direction to the further immune response. Two studies that examined the role of Gal-3 in bone-marrow-derived dendritic cells found that Gal-3 reduces the secretion of IL-10 and IL-23, resulting in a negative effect on Th17 polarization and the exacerbation of experimental autoimmune encephalomyelitis [42,43]. Studies indicate that Gal-3 induces NLRP3 (NLR family pyrin domain-containing 3) inflammasome activation in macrophages in a model of dextran sulfate sodium (DSS)-induced colitis, while macrophages of Gal-3 knock-out mice produce less TNF-α and IL-1β [44]. Pectic polysaccharide derived from Smilax China L. reduced symptoms of ulcerative colitis in animal models by inhibiting the galectin-3/NLRP3 axis [45]. In other experiments, it has been shown that Gal-3 promotes inflammation through the TLR4 (toll-like receptor 4)/MyD88 (myeloid differentiation primary response 88)/NF-κB (nuclear factor kappa B) pathway in microglia and in animal models of experimental autoimmune uveitis [46]. Macrophages are myeloid-derived cells characterized by great plasticity of and variation in phenotypes and function depending on the local cytokine milieu [47]. Gal-3 is shown to ameliorate IL-4-induced alternative polarization of bone marrow-derived macrophages, resulting in myocardial reparation and fibrosis after myocardial infarction [48]. Gal-3 is probably involved in renal fibrosis via the alternative polarization of macrophages toward M2, since Twist1 knock-out mice develop reduced renal fibrosis and Twist-1 is known to be a stimulator of Gal-3 expression [49]. Gal-3 seems to be involved in neurodegenerative diseases. In the mouse model of Huntington’s disease, intracellular aggregates induce lysosomal damage, while Gal-3 surrounds damaged lysosomes and induces inflammation through NF-κB and NLRP3 inflammasome-dependent pathways [50]. Experimental studies showed that NASH (nonalcoholic steatohepatitis) induced by a high-fat diet is characterized by higher TLR4 expression and NLRP3 inflammasome activation, while the Gal-3 inhibitor reduced liver inflammation and TLR4, NLRP3, and caspase-1 expression levels [51]. Modified citrus pectin (MCP), a competitive inhibitor of intra- and extracellular Gal-3, reduces inflammation by decreasing activation of the TLR4/NF-κB pathway and NLRP3 inflammasomes in microglia in a mouse model of cerebral ischemia/reperfusion injury [52].

## 4. Gal-3 and Tissue Fibrosis

Tissue fibrosis is the final stage of many chronic inflammatory reactions, resulting in extracellular matrix accumulation and tissue scaring and hardening, most commonly encountered in chronic renal, pulmonary, and liver diseases [53]. Tissue fibrosis can even lead to organ failure, and sometimes the only available therapy is organ transplantation [54]. Gal-3 is the key regulator of the process of tissue fibrosis by inducing fibroblast activation, ECM (extracellular matrix) deposition, and β-catenin pathway activation [55]. The antagonism of Gal-3 represents a potential therapeutic strategy for fibrotic diseases [56]. The role of Gal-3 in fibrosis of the liver, lungs, digestive system, skin, pancreas, and kidneys has been proven [57,58,59,60]. Gal-3-deficient mice have less prominent lung fibrosis, while Gal-3 is more abundantly expressed in the lung tissue of patients with idiopathic pulmonary fibrosis [55,61]. Gal-3 inhibition by TD139, a high-affinity galectin-3 inhibitor, attenuates β-catenin activation and reduces fibrosis in an animal model of bleomycin-induced lung fibrosis [55]. Gal-3-deficient mice also develop reduced renal fibrosis in a model of unilateral ureteric obstruction-induced fibrosis, with significantly decreased collagen tissue deposition and α-SMA (α-smooth muscle actin) mRNA expression [62]. Another study shows that increased Gal-3 expression is associated with cardiac fibrosis and risk of sudden cardiac death [63]. The inhibition of Gal-3 with MCP in an animal model of myocardial fibrosis results in the attenuation of cardiac function decline and a reduction in collagen deposition in cardiac tissue, associated with decreased expression of Gal-3, TLR4, and MyD88 and lower levels of NF-κB mediated inflammation [64]. A recent study shows that Gal-3 inhibition provides better results for the amelioration of cardiac inflammation induced by β-adrenergic receptor stimulation compared to β-blockers [65].

## 5. Harmful Effects of the Products of Lysed Erythrocytes

The intravascular lysis of erythrocytes leads to the release of many proinflammatory and pro-oxidative components directly into circulation, where the most prominent role are allotted to hemoglobin and its prosthetic group heme. Releasing hemoglobin and heme into circulation also leads to a decreased oxygen-carrying capacity, resulting in hypoxemia. The scavenger proteins haptoglobin and hemopexin neutralize hemoglobin and heme, preventing the induction of inflammation and organ damage. When intravascular hemolysis is extensive, the protective mechanisms of haptoglobin and hemopexin are exceeded, and heme and hemoglobin express their oxidative and damaging properties to organs such as the kidneys, liver, and spleen [5,66].

Cell-free hemoglobin is in a state of equilibrium between αβ heterodimers and tetramers, where hemoglobin dimers are capable of extravasation and pass through the renal glomerular membrane due to its relatively small size (31 kDa) [67]. Also, cell-free hemoglobin is in a constantly changing state when tetramers break down to dimers and ultimately release unbound or free heme, which is bound by circulating hemopexin and taken up by hepatocytes via the receptor CD91 [68]. In perivascular and subendothelial space, hemoglobin acts as a highly reactive molecule that induces cellular damage. The heme group of hemoglobin contains an iron atom in the reactive ferrous (Fe^2+^) state, which enables hemoglobin to act as an electron acceptor, bind diatomic gases, and react with physiological oxidants. Extracellular hemoglobin interacts with physiological oxidants such as hydrogen peroxide, which induces the transition of heme iron from a ferrous to a ferric hemoglobin (Hb-Fe^3+^), and highly reactive hydroxyl radicals, which trigger a cascade of redox reactions, resulting in extensive lipid peroxidation and cellular injury in the kidneys [69]. The reactive iron enables hemoglobin to bind nitric oxide (NO) in a reaction, whose main products are nitrate (NO_3_) and ferric hemoglobin (Hb-Fe^3+^) [70]. This reaction leads to NO depletion, resulting in endothelial dysfunction and mild hypertension due to vasoconstriction [71,72]. Vasoconstriction, in particular, affects renal hemodynamical function, and chronic hemolysis can lead to a lower glomerular filtration rate, which is commonly described in SCD [73,74]. Kidneys filtrate all the excess hemoglobin that cannot be scavenged by haptoglobin, and as a result, they are prone to hemoglobin-induced injury during hemolysis. Renal damage can lead to acute, but also chronic, kidney disease, leading to greater morbidity and mortality in chronic or massive hemolytic diseases [75]. Hgb-Fe^3+^ (methemoglobin) easily releases free heme, leading to secondary toxicity by heme’s oxidative potential. Heme is able, due to its lipophilic structure, to integrate into lipid membranes such as cell membranes and create free radicals that induce the lipid peroxidation of arachnoid acid and release of prostanoids with possible vasoactive properties [5]. Intravascular hemolysis and free heme occurrence induce the expression of HO-1, an inducible enzyme with antioxidative and anti-inflammatory capacities [76]. HO-1 degrades heme into biliverdin, CO, and labile iron [77]. Free heme also induces the endothelial activation and recruitment of leukocytes by enhancing the synthesis of adhesion molecules [78]. Heme is lipophilic and easily incorporates into the lipid membranes, where iron can catalyze the formation of cytotoxic lipid peroxide and enhances membrane permeability, leading to cell lysis and death [79,80,81,82]. Heme also catalyzes the reaction of the oxidation of low-density lipoprotein (LDL). Oxidized LDL contains toxic moieties that contribute to the activation of endothelia and inflammation induction [79].

Heme is known as a DAMP that activates innate immune response and inflammation, thus contributing to the damage of local tissues [7]. Macrophages bind heme via pattern recognition receptors, including TLR4 [83,84] and NLRP3 [85,86], and in response activate sterile inflammatory pathways. Labile heme enhances the expression of adhesion molecules such as intercellular adhesion molecule 1, E-selectin, and P-selectin in endothelial cells in various organs including the liver and kidneys [86,87], attracts neutrophils in vivo and in vitro [88,89], and induces the reorganization of the actin cytoskeleton, thus enabling the motile activities of neutrophils, migration and phagocytosis, and expression of IL-8. Further, heme triggers neutrophils to form neutrophil extracellular traps and, thus, can exacerbate organ damage, as demonstrated in SCD [90]. The expression of endogenous ligands for TLR4, such as HMGB1 (high-mobility group box 1) can be triggered by heme, and it, thus, enhances the production of proinflammatory cytokines in macrophages exposed to HMGB1 [91]. Hemoproteins induce the overexpression of TGF β1 (transforming growth factor-β isoform 1), which promotes fibrosis, leading to tubulointerstitial disease [92].

## 6. The Role of Gal-3 in Renal Injury Induced by Intravascular Hemolysis

Acute and chronic kidney injuries are complications of many diseases connected with intravascular hemolysis. The main pathway of hemoglobin clearance after saturation of protective plasma scavenger mechanisms is through the kidneys; hence, kidneys are the major organs that suffer after intravascular hemolysis due to exposure to free hemoglobin and heme [93]. Many mechanisms lead to renal injury and acute tubular necrosis during intravascular hemolysis, including heme and iron cytotoxic effects, oxidative stress and free radical generation, NO consumption that leads to vasoconstriction and decreased renal flow, the interaction of hemoglobin with Tamm-Horsfall protein, and the induction of inflammation [86,94]. The presence of heme alone induces apoptotic death in renal cells, as well as necroptosis and ferroptosis [95,96]. Free iron is also shown to be toxic to the renal cells due to its oxidative effect by inducing reactive oxygen species (ROS) [97]. Proinflammatory activities of heme greatly contribute to kidney injury after intravascular hemolysis. It has been documented that heme increases the expression of the chemokines CCL2 (chemokine (C-C motif) ligand 2) in renal tubular cells in vitro and in the kidneys in vivo, but it also enhances the expression of adhesion molecules in endothelial cells in the kidneys [86,87]. The activation of endothelia further promotes the influx of leukocytes into the vessel wall, leading to the further propagation of renal inflammation. Hemoglobin and heme released during PHZ-induced hemolysis in animals induce the activation of endothelia and subsequent inflammation and tubular injury [87]. Heme induces the activation of NLRP3-dependent inflammasomes in macrophages, followed by the secretion of proinflammatory IL-1β and sterile injury in animal models of intravascular hemolysis induced by unilateral ureteral obstruction [85]. The activation of NLRP3 inflammasome is also observed in human endothelial cells, resulting in the production of IL-1β [98,99]. As a DAMP, heme binds to TLR4 expressed on the cells of innate immunity, inducing a cellular signal cascade that finally activates NF-κB. Activated NF-κB can translocate to the nucleus and stimulates the expression of adhesion molecules and various proinflammatory cytokines, primarily tumor necrosis factor alpha (TNF-α) [84]. Heme also potentiates the stimulation of TLR4 with lipopolysaccharide [100]. TLR4 is also known as one of the main mediators of inflammatory response in acute renal ischemia/reperfusion injury [101]. Another study emphasizes the role of TLR4 in intravascular hemolysis induced by PHZ, where it is found that the absence/inhibition of TLR4 leads to the improvement of renal tubular damage and necrosis [102]. The inhibition of TLR4 attenuates pathological changes in the renal tubular epithelium and decreases NF-κB activation and IL-6, CCL2, and TNF-α expression [102]. In a mouse model of sickle cell anemia, it has been shown that heme activates the complement system and leads to C3 and C5b-9 deposition, while hemopexin attenuates this harmful effect [103]. Neutrophile infiltration in renal injury has previously been documented [104]. Heme alone is a potent chemoattractant for neutrophils [88]. Leukotriene B4 produced during hemolysis is a powerful stimulator of neutrophile recruitment and ROS production [105]. Heme and free iron also stimulate neutrophils to produce intracellular ROS and create neutrophil extracellular traps that can cause vaso-occlusion and eventually lead to the death of mice suffering from SCD [90,106].

Almost all mechanisms involved in the development of kidney injury after intravascular hemolysis can be affected by Gal-3 (Table 1). Also, the opposite roles of Gal-3 in the regulation of some of these mechanisms are reported (Table 1). The dual role of Gal-3 in the regulation of apoptotic cell death is well known and depends mainly on the localization of Gal-3. Gal-3 is released from erythrocytes after hemolysis and, thus, can modulate the pathological processes triggered by heme, iron, and hemoglobin that ultimately lead to kidney damage.

The role of Gal-3 in the pathogenesis of many renal diseases has been reported, along with potential beneficial effects of Gal-3 inhibition; however, there are no data about its possible role in kidney damage induced by acute intravascular hemolysis.

Several studies report the effects of Gal-3 on renal ischemia/reperfusion injury. In transitory renal ischemia induced in rats, increased expression of Gal-3 mRNA associated with a negative correlation with serum creatinine has been noticed 48 h after injury [119]. Another study reports increased expression of proinflammatory cytokines (IL-1β, IL-6, TNF-α, and IL-10) after renal ischemia/reperfusion injury, associated with enhanced expression of Gal-3 and increased plasma levels of Gal-3, which contribute to remote cardiac injury and even fibrosis [120]. The inhibition of Gal-3 by MCP decreased levels of proinflammatory cytokines and prevented acute kidney injury-induced cardiac damage and fibrosis [120]. In the experimental model of renal toxic nephropathy induced by folic acid, a transient increase in Gal-3 mRNA expression in many renal compartments, and also after 14 days in renal macrophages, is noticed and probably leads to renal fibrosis [119]. Gal-3 is shown to be a key molecule that renal macrophages secrete in response to renal injury in a model of unilateral ureteric obstruction, which promotes renal fibrosis by promoting fibroblasts to switch into a profibrotic phenotype [62]. Gal-3 again is shown to play an important role in the progression of renal injury caused by cisplatin, while Gal-3 inhibition reduces nephrotoxicity by decreasing renal fibrosis and apoptosis [121]. Contrary to this finding, another study has found that Gal-3 shows anti-inflammatory and protective effects in a similar model of cisplatin-induced renal injury by influencing dendritic cells to stimulate regulatory T-cell proliferation and IL-10 secretion [122]. A more recent study on cisplatin-induced kidney injury reports higher serum urea and creatinine levels, as well as higher levels of renal cell necroptosis, in Gal-3 knock-out mice compared to mice with normal Gal-3 expression [109]. These results indicate that Gal-3 can affect the inflammatory process both positively and negatively, depending on which cell and pathway is involved. In an experimental model of kidney hypertensive nephropathy, the inhibition of Gal-3 decreases the secretion of IL-6 and CCL2 and is protective against kidney injury [123]. Gal-3 is found to contribute to aldosterone-induced myocardial and renal fibrosis and organ dysfunction by NF-Kβ activation, which is reversed by Gal-3 inhibition in knock-out mice [124]. In another study, increased levels of Gal-3 after aldosterone stimulation are reported, followed by ECM deposition, while Gal-3 silencing reduces only aldosterone-induced, but not basal levels of, ECM deposition [57]. Gal-3 is also overexpressed in diabetic nephropathy and influences tissue remodeling [125].

**Figure 2:**
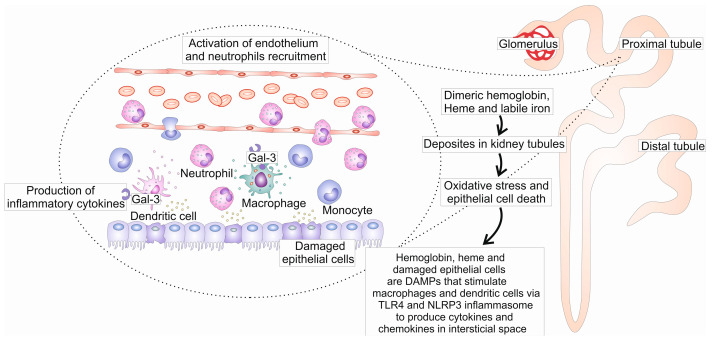
The possible role of Gal-3 in the development of renal damage induced by intravascular hemolysis. Severe hemolysis is accompanied by the accumulation of hemoglobin, heme, and iron in the kidneys, resulting in oxidative stress-mediated cell death and acute kidney injury. Damaged cells, heme, and hemoglobin stimulate dendritic cells and macrophages to produce inflammatory cytokines, together with molecules released from erythrocytes activate endothelial cells. The activation of endothelium enhances the influx of inflammatory cells. Gal-3 could affect the activation of dendritic cells and macrophages, and subsequently the expression of adhesion molecules in endothelium, and thus it could exacerbate inflammation and subsequent kidney injury.

Considering the significance of Gal-3 for the function of cells of innate immunity, the modulation of signals from receptors for DAMPs and TLR4 and the induction of inflammatory response, bearing in mind that these processes are significantly involved in kidney damage induced by intravascular hemolysis and that serum levels of Gal-3 are elevated in the samples with hemolysis, as well as recognizing the importance of Gal-3 in tissue fibrosis development, it is conceivable that Gal-3 may play a role in the pathogenesis of intravascular hemolysis-induced kidney damage (Figure 2) and fibrosis [5,13,56].

## 7. The Role of Gal-3 in Liver Injury Induced by Intravascular Hemolysis

Since the liver is the main organ where hemoglobin and heme can be metabolized, this organ is often affected by massive or chronic hemolysis. Hemoglobin and heme can disrupt sinusoidal microcirculation, leading to hepatocellular necrosis and, eventually, cirrhosis [126,127]. Many patients with SCD suffer from liver injury, fibrosis, and cirrhosis [128]. It has been demonstrated that liver injury in these patients is caused mainly by intravascular hemolysis and the vaso-occlusive effects of heme, as well as by iron overload [16]. The intraperitoneal application of ferryl-hemoglobin induces the recruitment of macrophages and neutrophils and activation of NLRP3 inflammasome and caspase-1, leading to enhanced IL-1β production in the livers of treated mice and enhanced liver inflammation [129].

Gal-3 has proven to be involved in the pathogenesis of many liver diseases, including liver fibrosis. Gal-3 secreted by liver monocytes and macrophages in response to stimuli activates myofibroblasts and, thus, contributes to liver fibrosis [130]. Tissue-resident macrophages are in charge of sensing and reacting to potential injury and also are the biggest source of Gal-3 in the liver [131]. In response to Gal-3 secretion, hepatic stellate cells are activated and change phenotype into myofibroblast-like collagen-producing cells [132]. Gal-3 expression is increased in fibrotic liver, while the blockage of Gal-3 leads to decreased myofibroblast activation and attenuation of fibrosis [59]. Early studies showed that Gal-3-deficient mice are predisposed to liver steatosis, cirrhosis, and, eventually, hepatocellular carcinoma [133,134]. Later, in an animal model of NASH, significantly reduced liver inflammation with attenuated expression of TLR4 and NLRP3 has been reported in Gal-3-deficient mice in comparison to Gal-3-expressing mice [135]. Secretory Gal-3, by engaging the peroxisome proliferator activated receptor γ (PPARγ)/CD36 signaling cascade, can cause hepatic steatosis in mice fed on a high-fat diet [136]. In a swine model of NASH, the expression of the LGALS3 gene is upregulated during the transition from steatosis to steatohepatitis, and increased expression of LGALS3 may be used in the evaluation of disease progression [137]. In a model of autoimmune cholangitis, Gal-3 stimulates inflammation through NLRP3 inflammasome activation and the induction of IL-1β production [138]. The results of all these previous studies indicate that Gal-3 inhibition can reduce inflammatory processes and fibrosis in animal models of different liver diseases [139]. Gal-3 is also upregulated in liver cirrhosis and hepatocellular carcinoma (HCC) [41,140]. Serum levels of Gal-3 are higher in hepatitis, cirrhosis, and hepatocellular carcinoma, though the level of Gal-3 cannot predict HCC development in cirrhotic patients but may be useful for cirrhosis diagnostics [141]. In advanced cirrhosis, Gal-3 is upregulated and correlates with disease severity, but it also predicts post-transplantation infectious complications [142].

The expression of Gal-3 in liver tissue is greatly enhanced in almost all liver diseases [132,140,143]. Gal-3 plays one of the main roles in the development of various inflammatory liver conditions and the key role in liver fibrosis [144,145]. Since the main pathological processes in the livers of patients with massive intravascular hemolysis include inflammation, fibrosis, and even cirrhosis, it can be assumed that Gal-3 most probably also affects liver injuries induced by acute intravascular hemolysis.

## 8. Potential Therapeutic Effects of Gal-3 Inhibition

According to the central role of Gal-3 in the pathogenesis of inflammatory and fibrotic changes in various organs, Gal-3 inhibition is of great interest in new drug development. The therapeutic effect of Gal-3 inhibition has been examined both in animal models and a few clinical trials. In concanavalin A-induced hepatitis in mice, the Gal-3 inhibitor TD139 had a beneficial effect when given as a pretreatment, which resulted in lowering liver inflammation [146]. Another study has shown that Gal-3 inhibitor DAVANAT^®^ reduces cholangitis induced by *Novosphingobium aromaticivorans* [138] and DSS-induced colitis [44] by reducing the activation of NLRP3 inflammasome and expression of IL-1β in macrophages. GB1211, a highly selective, orally active Gal-3 inhibitor, showed good therapeutic potential in mouse models of CCl4-induced liver fibrosis and bleomycin-induced lung fibrosis [147]. GB1211 was selected as a clinical candidate for clinical study, at phase IIa, as a potential therapy for patients with Child Pugh B and C (NCT05009680).

Several reports of preclinical and clinical studies imply that Gal-3 inhibition improves renal function in several pathological conditions, justifying the development of multiple Gal-3 inhibitors [148]. The inhibition of Gal-3 by MCP attenuates acute and chronic renal injuries induced by cisplatin [121], the partial occlusion of ascending aorta, and a high fat diet [149]. Preclinical studies have shown that the Gal-3 inhibitor GCS-100 reduces fibrosis, while in a randomized, blinded, phase IIa clinical trial, it has been shown that GCS-100 has beneficial effects in patients with stage 3b or 4 chronic kidney disease (NCT01843790). It was reported that Gal-3 inhibition is associated with slightly reduced plasma levels of creatinine in patients with hypertensive cardiac complications and an increase in eGFR, suggesting the potential of usage of Gal-3 inhibitors in the treatment of renal injuries [150].

## 9. Conclusions

Inflammation in acute kidney injury, including injury induced by acute intravascular hemolysis, culminates in tubulointerstitial fibrosis, which contributes to the development of progressive kidney disease. Thus, heme-induced vascular inflammation, complement activation, activation of endothelia, and infiltration of leukocytes have the potential to induce chronic renal inflammation and renal failure. An organ that is also frequently injured by hemolysis byproducts is the liver. The prevention of inflammation induced by acute intravascular hemolysis could prevent subsequent fibrosis and the development of chronic kidney diseases and liver fibrosis. Considering the involvement of Gal-3 in almost all inflammatory processes activated during acute intravascular hemolysis, this molecule could be one of the possible therapeutic targets. Future studies are needed to determine the precise role of Gal-3 in the development of acute and chronic tissue injuries induced by acute intravascular hemolysis and, considering the previously shown therapeutic effects of its inhibition on inflammatory and fibrotic diseases, reveal Gal-3 as a possible therapeutic target in hemolysis-induced organ damage.

## Figures and Tables

**Table 1:** How Gal-3 can affect pathological mechanisms involved in hemolysis-induced kidney injury.

Mechanisms of Renal Injury during Intravascular Hemolysis	How Gal-3 Affects the Mechanism
Heme and iron induce apoptotic death, necroptosis, and ferroptosis in renal cells [95,96].	Extracellular Gal-3 induces apoptosis of T cells [107] and monocytes/macrophages [108]. Intracellular Gal-3 attenuates cisplatin-induced apoptosis and necroptosis of renal tubule cells [109].
Heme and free iron induce oxidative stress and consequent inflammation and cell death [97].	Inhibition of Gal-3 reduces oxidative stress, apoptosis, and gene expression of inflammatory molecules in the retinal pigment epithelium [110]. Gal-3 enhances oxidative stress and fibrosis in doxorubicin-induced cardiac disfunction [111]. Gal-3 attenuates oxidative stress and apoptotic cell death in doxorubicin myocardial injury [112].
Heme increases the expression of adhesion molecules in endothelial cells in kidneys [86,87]. The activation of endothelia further promotes the influx of leukocytes into the vessel wall, leading to further propagation of renal inflammation.	Gal-3 interacts with different adhesive molecules expressed on endothelial cells including integrins, cadherins, endoglin, and the melanoma adhesion molecule (CD146) [18]. Interaction with CD146 activates the release of inflammatory cytokines from endothelial cells and enhances inflammatory processes [113].
Heme induces the activation of NLRP3-dependent inflammasomes in macrophages, followed by the secretion of proinflammatory IL-1β and sterile injury [85].	Gal-3 is a known enhancer of inflammasome activation and, thus, affects the development of different diseases [51,114,115,116].
Heme binds to TLR4 expressed on the cells of innate immunity, inducing a cellular signal cascade that finally activates NF-κB, thus stimulating the expression of adhesion molecules and proinflammatory cytokines [84].	Gal-3 stimulates TLR4/NF-κB signaling in immune cells and enhances inflammation [46,117,118].

## Abbreviations

galectin-3	Gal-3
autoimmune hemolytic anemia	AIHA
sickle cell disease	SCD
paroxysmal nocturnal hemoglobinuria	PNH
heme oxygenase-1	HO-1
damage-associated molecular pattern	DAMP
carbon recognition domain	CRD
carbohydrate binding proteins	CBPs
NLR family pyrin domain-containing 3	NLRP3
dextran sulfate sodium	DSS
toll-like receptor 4	TLR4
myeloid differentiation primary response 88	MyD88
nuclear factor kappa B	NF-κB
nonalcoholic steatohepatitis	NASH
modified citrus pectin	MCP
α-smooth muscle actin	α-SMA
low-density lipoprotein	LDL
high-mobility group box 1	HMGB1
reactive oxygen species	ROS
tumor necrosis factor alpha	TNF-α
chemokine (C-C motif) ligand 2	CCL2
phenylhydrazine	PHZ
peroxisome proliferator activated receptor γ	PPARγ
hepatocellular carcinoma	HCC
